# Gestion en urgence d’un énorme goitre compressif: à propos d’un cas

**DOI:** 10.11604/pamj.2022.41.265.30516

**Published:** 2022-03-31

**Authors:** Anajar Said, Coulibaly Konimba, Tahiri Ilias, Taali Loubna, Hajjij Amal, Saadi Mustapha, Ouraini Salwa, Jahidi Ali, Zalakh Mohammed, Benariba Fouad

**Affiliations:** 1 Ear, Neck, Throat Department, Face and Neck Surgery, Hospital Cheikh Khalifa, Mohammed VI University of Health Sciences, Casablanca, Morocco,; 2 Ear, Neck, Throat Department, Face and Neck Surgery, Military Hospital Mohammed V, Rabat, Morocco

**Keywords:** Goitre compressif, thyroïdectomie, dyspnée, cas clinique, Compressive goitre, thyroidectomy, dyspnea, case report

## Abstract

Le goitre plongeant compressif est une urgence au traitement du fait du risque d´asphyxie par compression de l´arbre respiratoire. Nous rapportons le cas d´une patiente de 48 ans qui s´est présentée aux urgences avec une dyspnée laryngée sur un goitre plongeant et compressif, à travers notre observation et une revue de la littérature nous allons mettre le point sur les caractéristiques cliniques, l'aspect radiologique, et les différentes options du traitement. La gestion d´un patient qui présente un goitre compressif est difficile et doit être rapide car le risque d´asphyxie est majeur, la prise en charge est multidisciplinaire par une équipe opératoire expérimentée incluant les réanimateurs l´Otho-rhino-laringologie (ORL) et les chirurgiens thoraciques.

## Abstract (en)

Compressive goitre is a public health emergency due to the risk of asphyxia caused by compression of bronchial tree. We report the case of a 48-year-old female patient presenting to the emergency department with laryngeal dyspnea due to compressive goitre. We conducted a study and a literature review focusing on the clinical and radiological features of compressive goitre and different treatment options. The management of a patient with compressive goitre is difficult and must be rapid, due to the high risk of asphyxia. It requires a multidisciplinary approach of an experienced surgical team, including ENT and thoracic surgeons.

## Introduction

Le goitre est défini comme une glande thyroïde pesant plus de 20 à 25 g avec un volume supérieur à 19 ml chez la femme et 25 ml chez l´homme [1,2]. Le goitre multinodulaire (MNG) est une entité pathologique clinique caractérisée par une augmentation du volume de la glande thyroïde avec formation de nodules [3]. Le goitre plongeant peut provoquer des symptômes de compression touchant la trachée, le larynx l´œsophage et le nerf laryngé récurrent. Ces symptômes sont généralement associés à des goitres malins et les goitres nodulaires bénins ne provoquent normalement pas de symptômes obstructifs [4].

## Patient et observation

**Information du patient**: il s´agit d´une patiente de 48 ans, qui s´est présentée aux urgences avec un goitre volumineux occupant toute la face antérieure du cou, évoluant depuis 20 ans qui est devenu compressif depuis 3 mois avec une dyspnée laryngée (la saturation de l'oxygène à l'air ambiant au repos à 88%) ayant nécessité le recours à l´oxygénothérapie par ailleurs, la patiente a rapporté une dysphagie au solide mais sans dysphonie associée.

**Résultats cliniques**: l´interrogatoire n'a pas révélé d'antécédents familiaux de goitre, ni d'irradiation dans l'enfance. L'examen clinique a trouvé un énorme goitre occupant toute la région antérieure du cou plongeant au niveau du médiastin sans adénopathies. La patiente était en euthyroïdie clinique et biologique (TSH et FT4 normaux).

**Figure 1:**
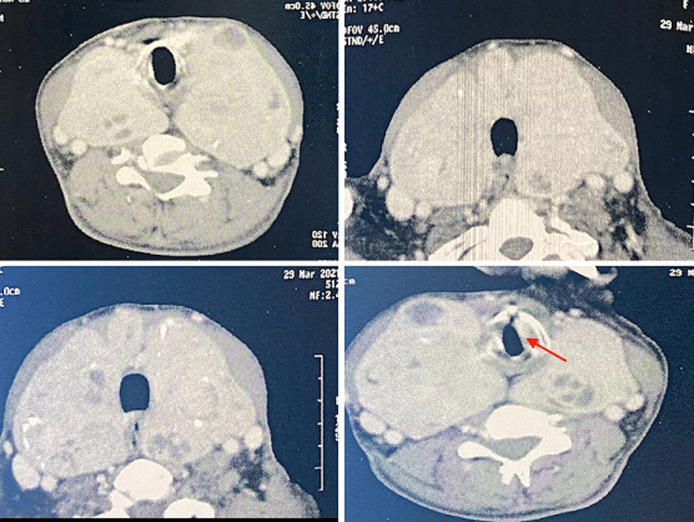
image scannographique du goitre compressif, la flèche montre la compression laryngée

**Démarche diagnostique**: le scanner cervicothoracique a objectivé un énorme goitre multi-nodulaire plongeant en intra-thoracique (Figure 1), comprimant sans envahir de façon régulière le larynx et la trachée.

**Figure 2:**
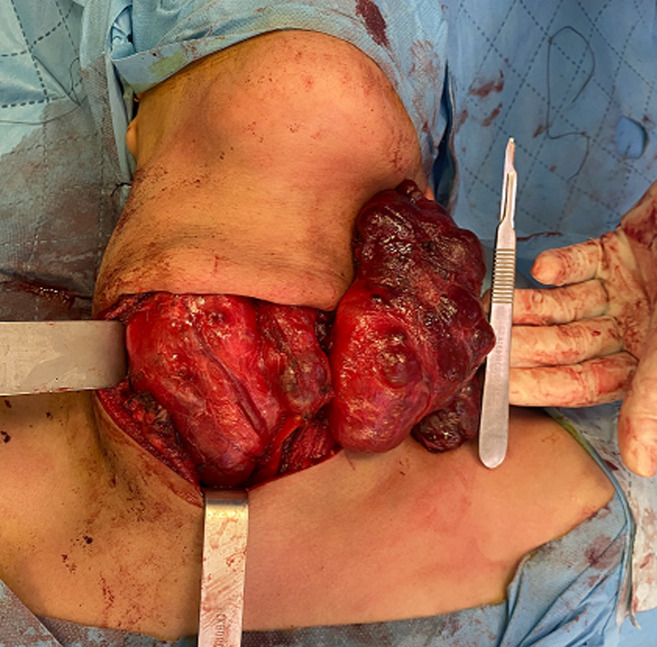
image peropératoire du goitre plongeant

**Intervention thérapeutique et suivi**: la patiente a été acheminée rapidement au bloc, bénéficiée d´une thyroïdectomie totale en urgence qui s´est déroulée sans incidents, les suites étaient simples, et les contrôles à 6 mois sont sans particularité (Figure 2).

**Consentement du patient**: il a été obtenu après explication et garantie de l´anonymat.

## Discussion

Le goitre multinodulaire (MNG) est une entité pathologique clinique caractérisée par une augmentation du volume de la glande thyroïde avec formation de nodules. Les nodules peuvent être très petits, souvent de quelques millimètres seulement, ou peuvent être plus gros, peut-être plusieurs centimètres chacun [5]. La glande thyroïde normale pèse environ 20 g, mais le poids de la glande varie en fonction du poids corporel et de l'apport en iode [6].

Le goitre multinodulaire peut être toxique ou non toxique et en cas de goitre multinodulaire toxique, le patient présente davantage des signes et des symptômes cardiovasculaires qui incluent des palpitations, une fibrillation auriculaire et d'autres tachyarythmies. En revanche, le goitre multinodulaire non toxique présente davantage de symptômes de compression [6,7]. La malignité dans certaines études montre qu'elle se produit à un taux allant jusqu'à 30%, et les facteurs de risque de malignité sont le sexe masculin, les petits nodules, un âge plus jeune. La chirurgie procure un soulagement instantané des effets de compressions, le cas de notre patiente qui a bénéficié d´une thyroïdectomie totale, aucune complication n'a été signalée et les principales complications auxquelles il faut s'attendre face à des goitres aussi énormes sont l'hémorragie lésion du nerf laryngé récurrent la trachéalmalacie et sa prise en charge qui doit être préparée. L'anesthésiste doit anticiper une intubation difficile et l'intubation par fibre optique comme était dans notre cas est le meilleur choix lorsqu'elle est disponible et lorsqu'elle n'est pas disponible, l'anesthésiste et le chirurgien doivent se préparer à une trachéotomie d'urgence en cas d'échec de l'intubation.

## Conclusion

Le goitre plongeant compressif est une urgence au traitement du fait du risque d´asphyxie par compression de l´arbre respiratoire, dans notre cas ni la mise en condition ni l´oxygénothérapie ni la corticothérapie n´a règlé le problème, nécessitant ainsi le recours à un traitement chirurgical en urgence.
